# A Protamine Knockdown Mimics the Function of *Sd* in *Drosophila melanogaster*

**DOI:** 10.1534/g3.120.401307

**Published:** 2020-04-22

**Authors:** Luke F. Gingell, Janna R. McLean

**Affiliations:** Department of Biology and Chemistry, Bethel University, Mishawaka, Indiana, 46545

**Keywords:** Segregation Distortion, Meiotic drive, RanGAP/GEF signaling, spermatogenesis, histone-to-protamine transition

## Abstract

*Segregation Distorter* (*SD*) is an autosomal meiotic drive system found worldwide in natural populations of *Drosophila melanogaster*. This gene complex induces the preferential and nearly exclusive transmission of the *SD* chromosome in *SD*/*SD^+^* males. This selfish propagation occurs through the interplay of the *Sd* locus, its enhancers and the *Rsp^s^* locus during spermatid development. The key distorter locus, *Sd*, encodes a truncated but enzymatically active RanGAP (RanGTPase-activating protein), a key nuclear transport factor in the Ran signaling pathway. When encoded by *Sd*, RanGAP is mislocalized to the nucleus interior, which then traps Ran inside the nucleus and disrupts nuclear import. As a result of this aberrant nuclear transport, a process known as the histone-to-protamine transition that is required for proper spermatid condensation fails to occur in *SD*/*SD*^+^ males. In this process, sperm-specific protamine proteins enter the spermatid nucleus and replace the formerly chromatin-complexed histones. Previously, we have shown that mutations affecting nuclear import and export can enhance distortion in an *SD* background, thus verifying that a defect in nuclear transport is responsible for the unequal transmission of chromosomes. Herein, we show that specifically reducing protamines induces distortion in an *SD* background, verifying that protamines are transported via the RanGAP/GEF pathway and indicating that *E(SD)* plays a significant and unique role in the process of distortion.

In the majority of cases, alleles at a given locus are represented in an organism’s offspring at a 1:1 ratio, however there do exist instances that do not adhere to this standard law of inheritance (Silver 1993; Yang *et al.* 2012). One of the best characterized instances is an autosomal meiotic drive system in *Drosophila melanogaster* known as *Segregation Distorter* (*SD*). This naturally occurring gene drive system is found in low frequencies (1–5%) on chromosome two in wild populations of *Drosophila* worldwide (Hiraizumi and Nakazima 1967; Hartl 1975; Hiraizumi and Thomas 1984; Temin and Marthas 1984). *SD* produces distorted allelic frequencies through its interference with spermatid maturation. During spermatogenesis in males heterozygous for *SD* (*SD*/*SD*^+^), most *SD*^+^-bearing spermatid nuclei fail to properly condense and thus are unable to undergo elongation and fertilization (Tokuyasu *et al.* 1977). The observed asymmetrical segregation pattern subverts Mendel’s first law and causes *SD*/*SD^+^* male fruit flies to preferentially transmit the *SD* chromosome to nearly 100% of their offspring (Ganetzky 1977). This causes almost all the offspring from *SD*/*SD*^+^ males to inherit the *SD* phenotype (Kusano *et al.* 2002). Although it has been over fifty years since *SD* was first discovered and initially characterized, the molecular basis through which *SD* is able to monopolize transmission is not fully understood.

A complete mechanistic understanding of *SD* has been elusive to obtain due to the complex ways this coadapted multigene system interacts inside the *Drosophila* genome. The components of the *SD* system are located at various points along the *SD* chromosome and include the primary locus, *Sd*, along with several modifying elements known as *Enhancer of SD [E(SD)] Modifier of SD [M(SD)]* and *Stabilizer of SD [St(SD)]* (Ganetzky 1977; Hiraizumi *et al.* 1980; Brittnacher and Ganetzky 1984). Due to the most robust losses of distortion being observed in *Sd* knockouts, *Sd* has long been understood to be the most functionally vital gene in this selfish distorting system (Ganetzky 1977). However, *Sd* still requires the correct genetic background to elicit distortion. This background consists of the upward modifiers of *Sd* and can be understood collectively with *Sd* as the *SD* complex. The strength of this complex to distort increases in a dose dependent manner with the number of modifying elements (*i.e.*, when all the elements of the *SD* system are present, the highest levels of distortion are observed). *Sd* and its upward modifiers collectively exert their distorting influence only in the presence of their molecular target, *Responder* (*Rsp*), which corresponds to a sequence of repetitive satellite DNA in the proximal heterochromatin (Sandler and Hiraizumi 1960; Wu *et al.* 1988; Pimpinelli and Dimitri 1989). Mutants sensitive to the action of *Sd* and its modifiers possess *Rsp^s^* or *Rsp*^*ss*^ alleles, while insensitive, non-distorting mutants possess the *Rsp^i^* allele (Hartl 1975). Interestingly, the degree of sensitivity to distortion correlates with the number of *Rsp* repeats, as there are ∼2500 copies of *Rsp*^*ss*^, ∼700 copies of *Rsp^s^* and 100-200 copies of *Rsp^i^* that are associated with *SD^+^* chromosomes (Wu *et al.* 1988).

Although *SD* is understood to be an example of meiotic drive, distorting males proceed through meiosis normally. It is post-meiotically during spermatid condensation that the *SD* spermatid gains a transmission advantage over its homolog. Specifically a process known as the histone-protamine transition that takes place during normal spermatogenesis fails to occur in *SD*^+^ spermatids (Kettaneh and Hartl 1976; Rathke *et al.* 2007). In this process, the sperm-specific protamine proteins enter the spermatid nucleus and replace the previously chromatin-complexed histones, resulting in the formation of a highly compact chromatin structure (Doyen *et al.* 2013). The proteins replacing histones in the mature chromatin complex are Protamine A (Mst35Ba), Protamine B (Mst35Bb) and the histone H1-like linker protein Mst77F (Jayaramaiah Raja and Renkawitz-Pohl 2005). The exchanging of histones for these proteins causes the spermatid’s nuclear volume to become two orders of magnitude smaller than a non-dividing somatic cell and allows for subsequent spermatid elongation and maturation to occur (Doyen *et al.* 2013). This repackaging of the paternal genome is mediated by the histone chaperone CAF-1. This protein is essential to the conversion from a nucleosomal to a protamine-based chromatin composition as it is responsible for loading protamines into the transitioning chromatin structure (Doyen *et al.* 2013). Interestingly, Mst77F is thought to be required for male fruit fly fertility (Rathke *et al.* 2010), while Mst35Ba and Mst35Bb, although essential for proper nuclear sperm condensation, are not (Tirmarche *et al.* 2014).

*Sd* encodes a mutant form of the RanGAP protein, which lacks one of two nuclear export signals (Kusano *et al.* 2001). This mutant form of RanGap is enzymatically active, but mislocalized to the interior of the nucleus, rather than the cytoplasmic face (Merrill *et al.* 1999). The activation of cytosolic Ran’s GTPase activity by RanGAP is required for the transport of proteins from the cytosol into the nucleus. As a result, when RanGAP is mislocalized to the nucleus interior, normal nuclear import fails to occur (Kusano *et al.* 2001). Since the entrance of protamines into the nucleus is critical for proper spermatid development, we wanted to investigate whether a knockdown of protamine, as well as knockdowns of other proteins indicated in the histone-to-protamine transition, were capable of distorting allelic frequencies in the absence of *Sd*.

Herein, we sought to elucidate the underlying mechanisms of *SD*^+^ spermatid failure through examining the effects of knocking down proteins involved in chromatin reorganization. We found that Bj1(RCC1), CAF-1 and protamine knockdowns were all capable of eliciting distortion. Additionally, we found that Bj1(RCC1) and protamine knockdowns were able to achieve distortion values equivalent to those seen when the full complement of *SD* components are present. Therefore we suggest that i) the histone-protamine transition is the point in spermatogenesis where *SD* exerts its effects at the cytological level; ii) protamines are, as presumed, transported by the RanGAP/GEF pathway; iii) protamine depletion is a mechanism involved in *SD*^+^ spermatid failure; and iv) distortion requires an additional *E(SD)* function unrelated to RanGAP mislocalization and subsequent nuclear protamine depletion.

## Materials and Methods

### Genetic stocks and markers

Stocks were maintained on standard cornmeal molasses agar at 18°. Crosses were all performed at 25°. Unless otherwise stated, all stocks were obtained from the Bloomington Drosophila Stock Center. The protamine UAS-RNAi stocks were obtained from the Vienna Drosophila Research Center. The *bam-Gal4* stock was a gift from Dr. M. Fuller’s lab. Protamine deletions were a gift from Dr. B. Loppin.

### SD stocks

Most *SD* stocks were obtained from B. Ganetzky. *SD-5* is a strong distorting chromosome containing *Sd*, *E(SD)*, *Rsp^i^ M(SD)* and *St(SD)* (Sandler *et al.* 1959). *SD-5^r7^* is a revertant of *SD-5* with an *Sd^+^* allele (Ganetzky 1977). *Y-Rsp^s^B^s^* contains the *Rsp^s^* locus translocated onto the Y chromosome (Lyttle 1989). *Sd-125A* was obtained from Cindi Staber. It contains the *Sd* duplication inserted onto the X chromosome along with the *w^+^* mini-gene in a p-element. This element was mobilized and flies carrying the element on the 2^nd^ chromosome were obtained and tested for their ability to cause distortion before being used in subsequent experiments.

### k tests

*k* tests were performed according to McLean *et al.* (1994), except that frequently only 10 individual males were tested for each cross, and 2 virgin *cnbw* or *w* females were used. *k* values were determined as the number of *Rsp^i^*-bearing offspring divided by the total, and are shown as unweighted means ± 2 SE.

### Data availability

Strains are available upon request. The authors affirm that all data necessary for confirming the conclusions of the article are present within the article, figures, and tables.

## Results

### ProtamineRNAi and Bj1(RCC1)RNAi induce distortion in the presence of SD’s upward modifiers

Previous work demonstrates that distortion is caused by the presence of Sd-RanGAP in the nucleus, resulting in nuclear transport failure (Merrill *et al.* 1999; Kusano *et al.* 2001). Interestingly, Sd-RanGAP appears to only have an effect on developing sperm cells (Kusano *et al.* 2002). This suggests that some protein or proteins necessary for sperm development might be unable to enter the sperm nucleus in sufficient quantity to allow for the proper development of *Rsp*^s^-bearing spermatids. Previous work has identified the histone-protamine transition as a possible stage of spermatogenesis that is affected by distortion (Hauschteck-Jungen and Hartl 1982). Therefore, we chose to explore the effects of reducing proteins involved in this conversion.

RNAi knockdowns of several different proteins involved in chromatin reorganization were crossed into flies containing the *SD* revertant, *SD5^r7^*, and *Rsp*^s^. *SD5^r7^* contains all of the genetic components of *SD* except for the mutant *Sd* gene, and therefore, cannot induce distortion on its own (Ganetzky 1977). The results of the crosses were quantified by k tests and are shown in Table 1. CAF-1 mutants only elicited mild distortion that was significantly different from control values, but not equivalent to *Sd*-induced distortion. Both the Mst77f and protamine knockdowns induced low level distortion in the absence of the *SD* modifiers, however when these modifiers were added only the ProtamineRNAi mutants were found to have distorted allelic frequencies similar to those observed with a full *SD* chromosome. This finding implies that the basal levels of distortion induced by the Mst77f construct must be occurring through an *Sd*-independent pathway. Taken together these data suggest that protamine is the vital protein involved in spermatid condensation that is unable to enter the *SD*^+^ spermatid nucleus and whose nuclear depletion induces distortion, strongly tying together the relationship between mutant RanGAP, protamines and the failure of *SD*^+^ spermatid maturation.

**Table 1:** Distortion caused by RNAi knockdowns of genes involved in sperm-specific chromatin condensation

	k values	k values	k values
UAS-RNAi construct	Construct with no driver and no *SD* modifiers	Construct with driver, no *SD* modifiers	Construct, driver, and *SD* modifiers
Protamine A	0.528 ± 0.016	0.673 ± 0.056*^a^*	0.926 ± 0.017*^b^*
Protamine B	0.577 ± 0.023	0.760 ± 0.020*^a^*	0.888 ± 0.050*^b^*
Caf1-180	0.572 ± 0.032	0.578 ± 0.024	0.775 ± 0.043*^c^*
Mst77f*^d^*	0.502 ± 0.017	0.675 ± 0.082*^a^*	0.747 ± 0.058
Bj1(RCC1)	Undetermined	0.645 ± 0.044	0.956 ± 0.052*^b^*

a significant difference between with and without construct.

b significant difference from control and distortion levels are similar to that observed with a full *SD* chromosome.

c significant difference from control, but distortion levels are lower than those observed with a full *SD* chromosome.

d *α-tubulinGal4* driver; all others were *bamGal4* driver.

Aberrant RanGAP/GEF signaling is further supported as a causal agent in *SD* by the high levels of distortion observed in the Bj1(RCC1) knockdowns (Table 1). Bj1(RCC1) encodes RanGEF, which exchanges RanGDP for RanGTP inside the nucleus. This exchange is necessary to achieve proper nuclear transport, as only RanGTP can exit the nucleus and transport additional nuclear-bound cargo from the cytosol (Macara 2001). Consequently, knocking down the transcript for RanGEF decreases the ratio of nuclear RanGTP:RanGDP, trapping Ran in the nucleus and diminishing its protein transport capacity. *Sd*-RanGAP causes a similar nuclear confinement of Ran through its ability to activate Ran’s GTPase activity, which likewise results in a reduced RanGTP:RanGDP ratio in the nucleus. Therefore, this aberrant nuclear accumulation of Ran is likely impairing nuclear import of protamines by the RanGAP/GEF pathway and that could be inducing the observed distorted allelic frequencies. Thus, the *SD* phenotype production from the protamineRNAi-containing mutants suggests that ProtamineRNAi mimics the molecular action of *Sd* when in the presence of the modifiers of *SD*.

Previously it has been shown that deleting genes involved in nuclear transport enhance distortion and that the gene with the strongest ability to enhance distortion values, *embargoed*, is involved in Ran-dependent nuclear transport (McElroy *et al.* 2008). This study in tandem with our current finding that ProtamineRNAi induces distortion, further suggests that the nuclear import of protamines occurs through the Ran GAP/GEF system. This confirmatory finding transforms *SD* from an esoteric puzzle into a fascinating new medium through which the physiologically relevant Ran signaling pathway, and nuclear transport in general, may be studied.

### Two copies of Sd are insufficient to elicit distortion

Since no mutation in the *SD* system causes distortion alone, we were curious if two copies of *Sd* might cause distortion when separated from the other elements of the *SD* chromosome. This interest in evaluating the distorting ability of two doses of *Sd* stemmed from a finding that two copies of *E(SD)* are capable of eliciting distortion, while two copies of *Sd* are not (Temin 1991). This, coupled with the finding that the presence of *E*(*SD*) is required for full distortion, suggests that *E(SD)* is having some effect on *Sd*-RanGAP. Kusano *et al.* (2002) hypothesized that the molecular action of *E(SD)* is required for *Sd*-RanGAP to become mislocalized. This hypothesis is supported by the observation that without *E*(*SD*) only a minor amount of distortion occurs (Ganetzky 1977) and by the finding that *Sd* alone has very little distorting ability (McLean *et al.* 1994). We revisited the two doses of *Sd* mutants because those previously tested were recombinants of whole *SD* chromosomes, which could have allowed for possible lethal interactions between other associated alleles. To control for this, the mutants we tested were generated through the mobilization of a p-element containing the P^w+^*^Sd^* construct that had been inserted into an X chromosome (*Sd-124A*). To ensure that both copies of P^w+^*^Sd^* were viable producers of *SD* phenotypic ratios, each was individually tested for its ability to distort in the presence of the *SD* upward modifiers in the form of *SD*5^r7^. Distortion was measured using the Y-linked *Rsp^s^* allele. Upon confirmation of the P^w+^*^Sd^* construct’s distorting ability, we generated mutants carrying both of the *Sd* containing p-elements and tested their ability to distort without the associated *SD* loci present. We found that the presence of two copies of the P^w+^*^Sd^* construct did not significantly affect the transmission of the *Rsp^s^* allele (Table 2). This result provides strong support for the hypothesis that the additional elements of the *SD* chromosome, *i.e.*, the *SD* upward modifiers, are required for producing the high distortion ratios associated with *SD*.

**Table 2:** Multiple copies of *Sd* do not cause distortion alone

*Sd* Element	*SD* Complex	k value
*Sd-5A* (P^w+^*^Sd^* on 2^nd^ chromosome)	*SD5^r7^*	0.958 ± 0.019
		n = 437
*Sd-124A* (P^w+^*^Sd^* on 1^st^ chromosome)	*SD5^r7^*	0.996 ± 0.003
		n = 306
*Sd-5A* and *Sd-124A*	none	0.552 ± 0.012
		n = 973

n = the number of offspring counted.

### Protamine knockdowns and knockouts are unable to elicit distortion without SD’s upward modifying elements

If distortion is caused entirely by a lack of nuclear protamines, then a knockdown of protamines should alone be able to ellicit distortion without any additional *SD* elements. To test this, mutants possessing two copies of ProtamineRNAi were generated and their ability to distort was observed. The observed allelic frequencies for the ProtamineRNAi homozygotes did not significantly differ from those observed during normal segregation (Table 3). To further confirm that no amount of protamine reduction is able to independently induce distortion, mutants possessing two copies of protamine knockouts were also tested. In alignment with our previous prediction, distorted allelic frequencies were not observed (Table 3). This finding undermines the idea that differences in *Rsp^s^*/*Rsp*^i^ spermatid viability are completely due to differences in protamine requirement at the *Rsp* loci.

**Table 3:** Complete reduction in protamine alone is unable to cause distortion

Genotype	k value
*UAS-protARNAi/UAS-protBRNAi*; *αtubGal4*	0.674 ± 0.039
	n = 642
*Δmst35b/Δmst35bSco*	0.552 ± 0.025
	n = 517

n = the number of offspring counted.

### Sd does not distort with a protamine knockdown alone

Since *Sd* and ProtRNAi are incapable of eliciting distortion in both singlet and duplicate forms on their own, we tested whether some interaction between the two could cause distortion. Mutant constructs were generated that possessed one copy of the *Sd* containing p-element, P^w+^*^Sd^*, and one copy of ProtamineRNAi. Again, we found that when the *SD* upward modifiers are absent, genetic constructs that would otherwise distort allelic frequencies fail to do so (Table 4), suggesting distortion cannot be entirely explained by aberrant Ran signaling.

**Table 4:** Knockdown of protamine in conjunction with *Sd* is unable to cause distortion

Genotype	k value
*Sd-124A*; *UAS-protARNAi/+*; *αtubGal4*	0.633 ± 0.065
	n = 654
*Sd-124A*; *+*; *+*	0.630 ± 0.135
	n = 283

n = the number of offspring counted.

## Discussion

The aim of this study was to investigate the effects of knocking down proteins involved in the process of chromatin reorganization. Of the knockdowns tested in an *SD* background, only ProtamineRNAi and Bj1(RCC1)RNAi mutants were capable of distorting at a similar level to those where the full complement of *SD* elements were present. This confirms that protamines are necessary for proper spermatogenesis and suggests that a nuclear deficiency in protamine underlies the failure of the *SD*^+^ spermatid to undergo a histone-to-protamine transition. Additionally, the high levels of distortion observed in Bj1(RCC1) knockdowns supports the assertion that protamines are transported by the RanGAP/GEF pathway. McElroy *et al.* (2008) found that deleting genes involved in nuclear transport enhances distortion and that the deletion most able to enhance distortion was for the gene, *embargoed*, which is implicated in nucleocytoplasmic transport (Quimby and Dasso 2003; Di Fiore *et al.* 2004). Our current study, in tandem with the findings from McElroy *et al.* (2008), suggest that the molecular underpinnings of distortion involve aberrant Ran-signaling impairing nuclear protamine import, which then inhibits the establishment of a protamine-rich chromatin complex and induces *SD*^+^ spermatid failure.

The discovery that *Sd* encodes Sd-RanGAP, a truncated form of the normally encoded RanGAP, led to the development of the nuclear transport model as a possible explanation for the molecular operations of the *SD* system. This proposal relies on the known function of wild-type RanGAP in the transport of cytosolic cargo across the nuclear membrane. This ability requires RanGAP to be both enzymatically active (*i.e.*, capable of hydrolyzing Ran-GTP to Ran-GDP) and localized properly (*i.e.*, tethered to a nuclear pore on the cytoplasmic side of the nuclear envelope). The proper localization of RanGAP is of critical importance in the RanGAP-RanGEF system because without the proper RanGTP gradient (high in the nucleus and low in the cytoplasm) the directionality of transport into and out of the nucleus is lost (Macara 2001).

The nuclear transport model, first proposed by Kusano *et al.* in 2001, provides a viable explanation of the molecular mechanisms involved in *SD*. However, our findings complicate the predicted mechanism. If a loss of normal nuclear transport is the crux of why the *SD*^+^ spermatid fails to condense, and protamines are the nuclear cargo vital to spermatid condensation, then ProtRNAi mutants lacking the *SD* elements should elicit distortion, and yet they did not. The mutants able to distort were those possessing the upward modifiers of *Sd*. This suggest that one or all of the *SD* upward modifiers are required for distortion.

*E(SD*) may be responsible for increasing Sd-RanGAP’s ability to enter or remain in the nucleus (Kusano *et al.* 2002). This was first hypothesized after the discovery of wild-type RanGAP, when in the presence of two copies of *E*(*SD*), within the nucleus of primary spermatocytes (Kusano *et al.* 2002). *E(SD*)’s role in RanGAP mislocalization is further supported by its ability to induce low levels of distortion when present in two copies (Temin 1991) and the finding that *E(SD)* deletions drastically reduce distortion values (Ganetzky 1977; Brittnacher and Ganetzky 1984; Sharp *et al.* 1985). Additionally, Temin found that *Sd* and *E(SD)* are both suppressible by the same genetic modifiers and hypothesized that these two distorting constructs skew allelic frequencies through a shared pathway (Temin 1991). The fact that the ProtRNAi mutants fail to elicit distortion indicates that if these two genes share a pathway, the action of *E(SD)* must come subsequent to the action of *Sd*, not before it, or more likely *E*(*SD*) has a role secondary to causing the mislocalization of *Sd*. Impaired nuclear transport is certainly a piece of the *SD* puzzle, as deleting proteins involved in nuclear transport elicits distortion (McElroy *et al.* 2008). However, our findings suggest that *E(SD)* may have an additional function that is required for distortion, as neither knocking out the protamine transcript or reducing it in the presence of mutant RanGAP result in preferential transmission of *Rsp^i^*. Therefore, we propose that it is the E(SD)-assisted nuclear mislocalization of Sd-RanGAP in conjunction with some additional E(SD) function that prohibits *Rsp^s^*/protamine interaction and induces distortion. The findings herein shed light on the caveats to the nuclear transport model and demonstrate that more investigation into the role of E(SD) is necessary before a complete mechanistic determination can be obtained for this complex gene drive system.
